# Imipenem and relebactam pharmacokinetics in peritoneal fluid: microdialysis reveals infection-independent penetration

**DOI:** 10.1128/aac.01838-25

**Published:** 2026-07-31

**Authors:** Huan Tong, Zekun Cao, Jiakai Li, Haoming Yang, Yong Qiao, Siwei Guo, Changhui Deng, Xin Li

**Affiliations:** 1 Hunan Provincial Key Laboratory of Anti-Resistance Microbial Drugs, Department of Pharmacy, The Third Hospital of Changsha 610055, Changsha, People's Republic of China; 2 Hunan University of Chinese Medicine 118393 , Changsha, People's Republic of China; University of Pennsylvania Perelman School of Medicine, Philadelphia, Pennsylvania, USA

**Keywords:** microdialysis, imipenem, relebactam, intraperitoneal infection, pharmacokinetics

## Abstract

Imipenem/relebactam is a promising therapy for complicated intra-abdominal infections caused by carbapenem-resistant *Enterobacterales* species. However, data on tissue-site pharmacokinetics are limited. We evaluated the peritoneal penetration of imipenem and relebactam in healthy and infected rats by using microdialysis. The animals received a single intravenous infusion of imipenem (45 mg/kg) and relebactam (22.5 mg/kg), and unbound concentrations were dynamically monitored in the plasma and peritoneal fluid. Imipenem exposure in the peritoneal fluid was consistently lower than that in the plasma, with peritoneal fluid-to-plasma AUC ratios of 0.29 in the control group and 0.27 in the infection group. In contrast, relebactam demonstrated nearly equivalent concentrations in both compartments, with ratios of 0.81 and 0.86, respectively. Both agents reached peak levels at 30 min, and the semi-logarithmic curves showed rapid equilibrium without delay. The partition coefficients estimated by two-compartment modeling for imipenem (0.333–0.342) and relebactam (0.679–0.816) were consistent with non-compartmental results. Infection increased the distribution volume and clearance of relebactam but did not significantly alter peritoneal permeability for either drug. These findings provide the first description of the penetration of relebactam into peritoneal fluid. Subsequent pharmacodynamic target attainment (PTA) analysis showed that under the latest EUCAST PK/PD criteria, PTA values dropped markedly to an MIC of 1 mg/L, indicating a substantially reduced probability of achieving adequate peritoneal exposure. These results highlight the need for dosing strategies to ensure sufficient peritoneal drug concentration to maintain antimicrobial efficacy.

## INTRODUCTION

Complicated intra-abdominal infections (cIAIs), defined as infections within the peritoneal cavity that involve peritonitis or abscess formation, remain a major cause of morbidity and mortality in surgical and critically ill patients (1). These infections are primarily caused by gram-negative bacteria and anaerobes (2). Current treatment relies on broad-spectrum monotherapy or combination regimens, with β-lactam (BL) antibiotics forming the cornerstone of therapy (3, 4). However, the increasing prevalence of multidrug-resistant *Enterobacteriaceae*, particularly carbapenem-resistant organisms (CRO), has critically reduced the efficacy of BL (4). Combining BLs with β-lactamase inhibitors (BLIs) represents a key strategy for restoring the antimicrobial activity against resistant pathogens (5).

Relebactam is a novel diazabicyclooctane BLI that restores imipenem activity against CROs *in vitro* (5, 6). The fixed-dose combination of imipenem, cilastatin, and relebactam (marketed as Recarbrio) is approved for the treatment of moderate to severe cIAIs and represents a valuable therapeutic option in regions with a high prevalence of carbapenem-resistant pathogens (7, 8). Nevertheless, the clinical evidence supporting its use in cIAIs caused by CROs is limited. In the pivotal phase II trial that underpinned FDA approval, only 7 of 347 patients had infections caused by gram-negative organisms that were not susceptible to imipenem but susceptible to the imipenem/relebactam combination (9). Similarly, in a phase III multicenter study designed to target imipenem-resistant infections, only two patients with cIAI were enrolled, leaving a critical evidence gap regarding the clinical efficacy of imipenem/relebactam in CRO-associated cIAIs (10).

Since antibiotic efficacy depends not only on pathogen susceptibility but also on drug penetration into the site of infection, pharmacokinetic (PK) characterization of relevant compartments is essential. In intra-abdominal infections, bacteria commonly invade the mesentery, retroperitoneum, abdominal wall, and visceral organs; however, the peritoneal fluid represents the most critical extracellular compartment for evaluating antibacterial activity. Thus, a key determinant of the clinical effectiveness of BL/BLI combinations against CRO-associated cIAIs is whether both components achieve sufficient free concentrations in the peritoneal fluid, particularly whether the BLI reaches the levels necessary to restore the activity of the partner BL. To date, only imipenem concentrations in peritoneal fluid have been reported (11–14). Although relebactam concentrations in the epithelial lining fluid have been described (15), no data are available on its distribution in the peritoneal fluid.

Microdialysis is an established technique for assessing unbound antibiotic concentrations in interstitial fluids and has been widely applied to characterize tissue-site pharmacokinetics (16, 17). Compared with plasma concentration monitoring, microdialysis provides a more accurate representation of local drug exposure at the site of infection. In this study, we applied microdialysis in a rat model of peritoneal infection to characterize, for the first time, the peritoneal pharmacokinetics of imipenem and relebactam. Our aim was to determine whether infection alters peritoneal penetration and to evaluate the potential implications of optimizing dosing strategies for cIAIs.

## RESULTS

### Establishment and confirmation of the infection model

The *in vivo* recovery rates of the microdialysis probes were determined using a retrodialysis method. Probe recovery for imipenem ranged from 28% to 48% (mean ± SD: 35.6% ± 6.8%), whereas that for relebactam ranged from 16% to 44% (mean ± SD: 28.6% ± 9.6%). At 24 h post-infection, rats in the infection group exhibited typical clinical signs of systemic illness, including reduced activity, hunched posture, ruffled fur, and loose stools. Postmortem examination revealed greater omental adhesion, mesenteric vascular congestion, and turbid peritoneal effusion. Quantitative culture of peritoneal lavage fluid confirmed a bacterial burden of 5.8 ± 1.6 × 10^5^ CFU/mL, exceeding the predefined threshold and confirming successful model establishment.

**Fig 1:**
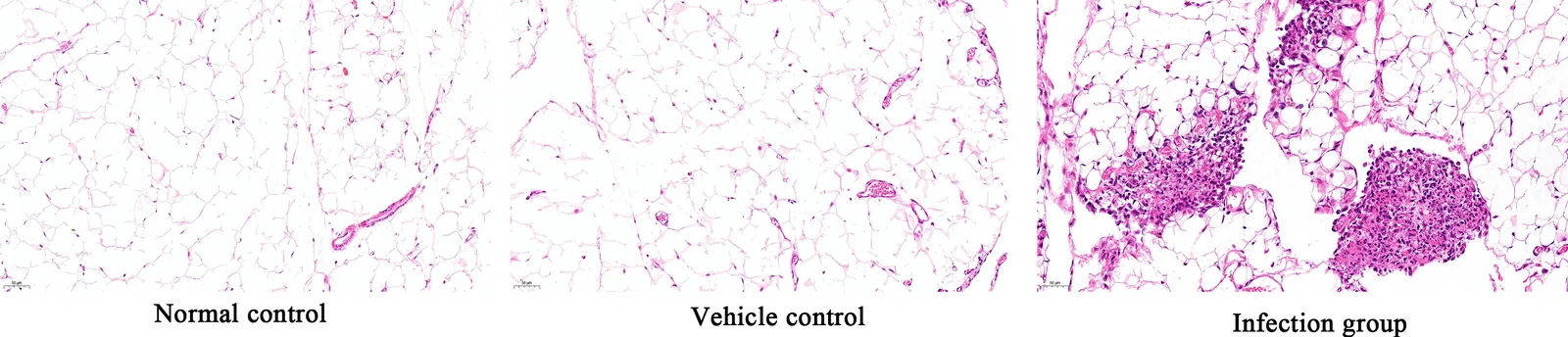
Histological photomicrographs of the greater omentum tissue sections stained with HE. Scale bar = 50 µm.

During the sampling process, mortality was observed in the infection group (3/9), and six animals were included in the final analysis. The histopathological analysis further supported these findings (Fig. 1). Hematoxylin and eosin (HE) staining of the greater omentum demonstrated marked infiltration of inflammatory cells, predominantly neutrophils and mononuclear cells, accompanied by disruption of adipose tissue architecture and accumulation of cellular debris and exudates. In contrast, the vehicle control group showed intact adipose tissue with sparse immune cell infiltration, whereas the normal control group showed no inflammatory changes. These findings indicate that the observed histological alterations were driven by the infection itself rather than by cyclophosphamide-induced immunosuppression.

### Pharmacokinetic analysis

**Fig 2:**
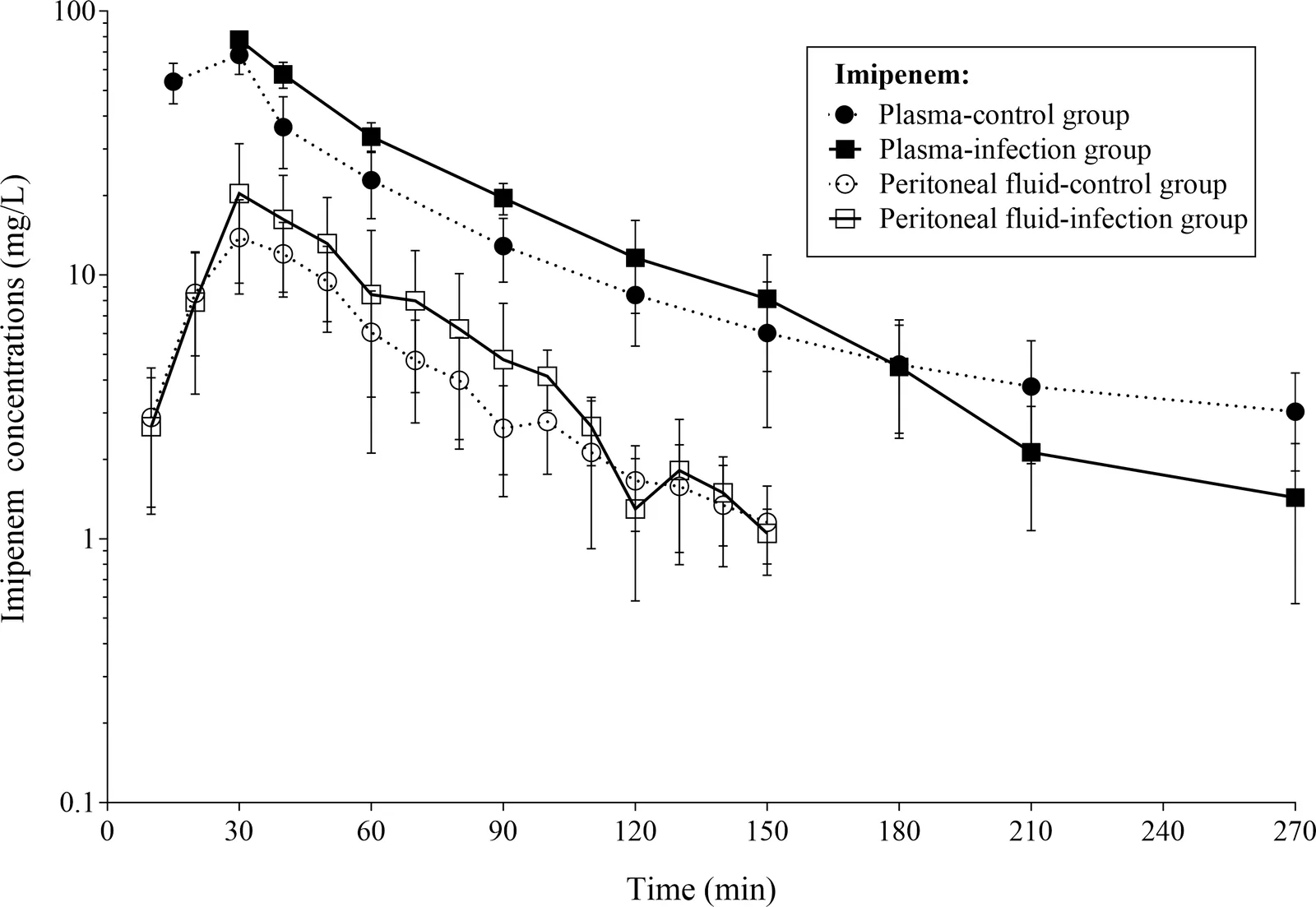
Unbound imipenem concentrations in plasma and peritoneal fluid following a single intravenous infusion of imipenem (45 mg/kg) and relebactam (22.5 mg/kg). Data are presented as mean ± SD for control and infected rats on a semilogarithmic scale.

**Fig 3:**
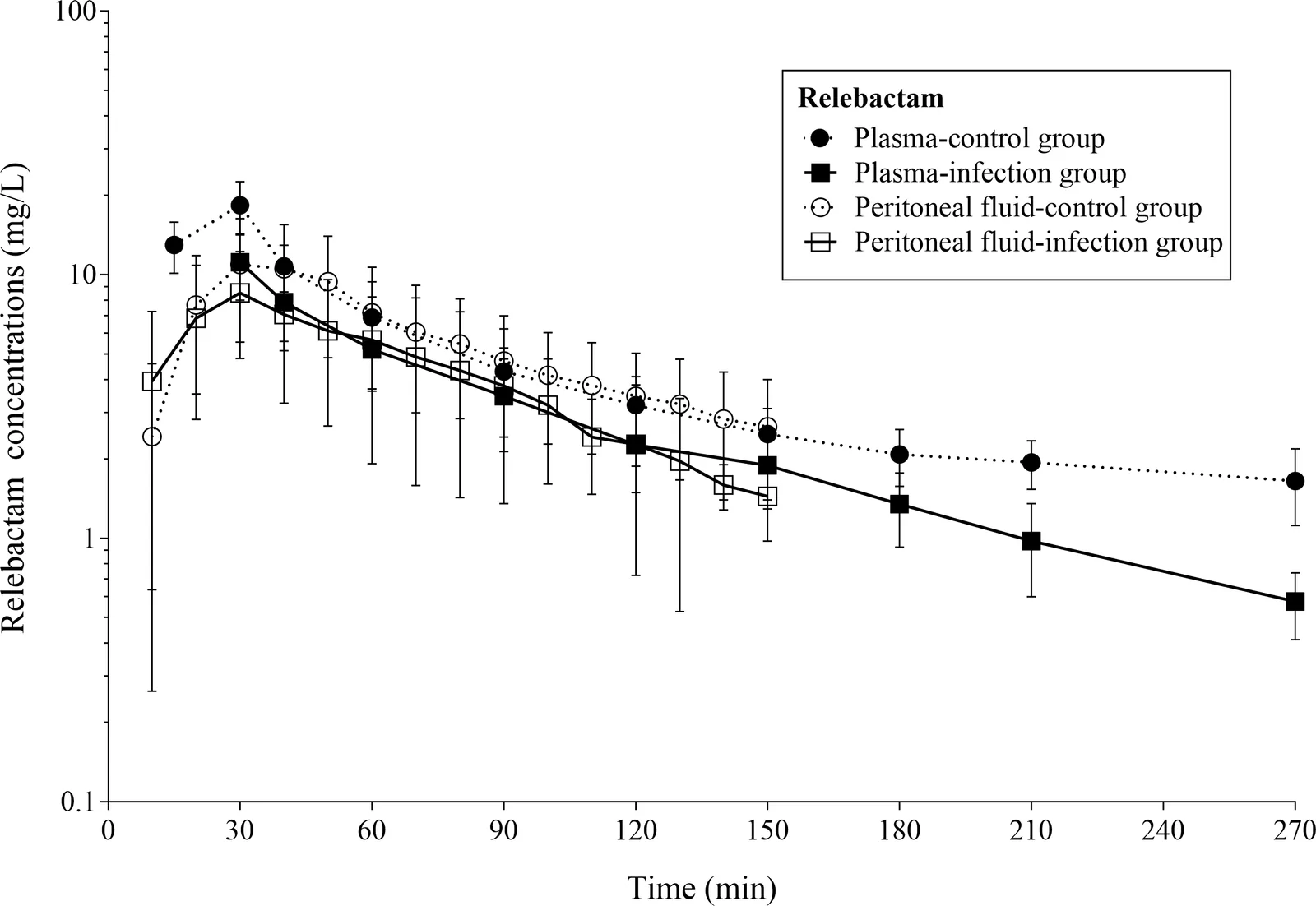
Unbound relebactam concentrations in plasma and peritoneal fluid following a single intravenous infusion of imipenem (45 mg/kg) and relebactam (22.5 mg/kg). Data are presented as mean ± SD for control and infected rats on a semilogarithmic scale.

Following intravenous infusion of imipenem (45 mg/kg) and relebactam (22.5 mg/kg), imipenem concentrations in the peritoneal fluid were consistently lower than those in the plasma (Fig. 2). The relative exposure, expressed as the peritoneal fluid-to-plasma AUC ratio, was 0.29 (95% CI: 0.17–0.41) in the control group and 0.27 (95% CI: 0.17–0.38) in the infection group. In contrast, relebactam concentrations in peritoneal fluid closely approximated those in unbound plasma (Fig. 3), with AUC ratios of 0.81 (95% CI: 0.47–1.15) and 0.86 (95% CI: 0.65–1.07), respectively (Table 1). No statistically significant differences were observed between the control and infection groups for either drug (imipenem, *P* = 0.41; relebactam, *P* = 0.53), supporting the conclusion that infection did not affect peritoneal penetration.

**TABLE 1:** Non-compartmental pharmacokinetic parameters (mean ± SD) of unbound imipenem and relebactam after a single intravenous infusion of 45 mg/kg imipenem and 22.5 mg/kg relebactam^*a*^

Sample type	PK parameter	Control group		Infection group	
		Imipenem	Relebactam	Imipenem	Relebactam
Plasma	*t*_1/2_ (min)	45.92 ± 10.86	75.42 ± 24.78	49.16 ± 16.94	73.36 ± 16.36
	*C*_max_ (mg/L)	54.50 ± 8.45	18.33 ± 4.18	62.40 ± 4.95	11.14 ± 3.13
	AUC_0–∞_ (mg/L·min)	3,279.87 ± 941.41	1,342.96 ± 252.47	3,835.25 ± 374.13	854.90 ± 267.44
Peritoneal fluid	*t*_1/2_ (min)	38.08 ± 5.11	60.05 ± 21.25	27.66 ± 2.79	43.55 ± 10.58
	*C*_max_ (mg/L)	14.23 ± 4.52	11.39 ± 5.26	21.47 ± 10.38	9.81 ± 4.63
	AUC_0–∞_ (mg/L·min)	883.73 ± 292.05	1,090.62 ± 489.56	1,022.02 ± 312.41	722.17 ± 274.53
Peritoneal fluid:plasma AUC_0–∞_ ratio		0.29 ± 0.11	0.81 ± 0.32	0.27 ± 0.10	0.86 ± 0.20

^*a*^ *t*_1/2_, the terminal elimination half-life; *C*_max_, observed maximum concentration; AUC_0–∞_, area under the concentration-time curve from zero to infinity.

Both agents reached peak concentrations ($t_{max}$) at 30 min. The arithmetic mean ratios of peritoneal fluid to plasma concentrations at each time point were highly consistent, and the semi-logarithmic concentration–time curves were nearly parallel (Fig. 2 and 3). For imipenem, the peritoneal fluid-to-plasma ratio ranged from 0.25 to 0.45 in the control group and 0.24 to 0.33 in the infection group. For relebactam, the ratio ranged from 0.59 to 1.03 and 0.80 to 1.02, respectively. These results demonstrate rapid equilibration between the plasma and peritoneal compartments with no observable distributional delay.

Model-based estimates aligned with the noncompartmental results. The partition coefficient (Kp) values for imipenem were 0.342 (control) and 0.333 (infection), whereas those for relebactam were 0.679 and 0.816, respectively (Table 2). The parameter precision was acceptable, and the two-compartment model demonstrated a high coefficient of determination (0.78–0.99), confirming the stability and reliability of the model.

**TABLE 2:** Pharmacokinetic parameters (mean ± SD) of unbound imipenem and relebactam after a single intravenous infusion of 45 mg/kg imipenem and 22.5 mg/kg relebactam based on the hypothetical two-compartment model^*a*^

Parameter	Control group		Infection group	
	Imipenem	Relebactam	Imipenem	Relebactam
*k*12 (1/min)	0.083 ± 0.024	0.057 ± 0.020	0.142 ± 0.160	0.208 ± 0.226
*k*21 (1/min)	0.046 ± 0.008	0.033 ± 0.008	0.075 ± 0.062	0.117 ± 0.166
*k*10 (1/min)	0.060 ± 0.017	0.035 ± 0.008	0.046 ± 0.031	0.046 ± 0.044
*V*_*c*_ (L/kg)	0.258 ± 0.049	0.899 ± 0.289	0.348 ± 0.161	1.574 ± 0.773
*V*_ss_ (L/kg)	0.723 ± 0.164	2.479 ± 0.871	0.620 ± 0.135	3.874 ± 1.352
CL (L/min/kg)	0.015 ± 0.003	0.029 ± 0.006	0.011 ± 0.001	0.038 ± 0.021
Kp	0.342 ± 0.124	0.679 ± 0.257	0.333 ± 0.138	0.816 ± 0.258

^*a*^ *k*12 and *k*21, transfer rate constants between the central and peripheral compartments; *k*10, elimination rate constant from the central compartment; *V_c_*, apparent volume of distribution in the central compartments; *V*_ss_, steady-state volume; CL, systemic clearance. Kp is the peritoneal-to-plasma partition coefficient, which is defined as the ratio of free drug concentration in the peritoneal fluid to that in the plasma.

### Peritoneal fluid PK/PD analysis

**Fig 4:**
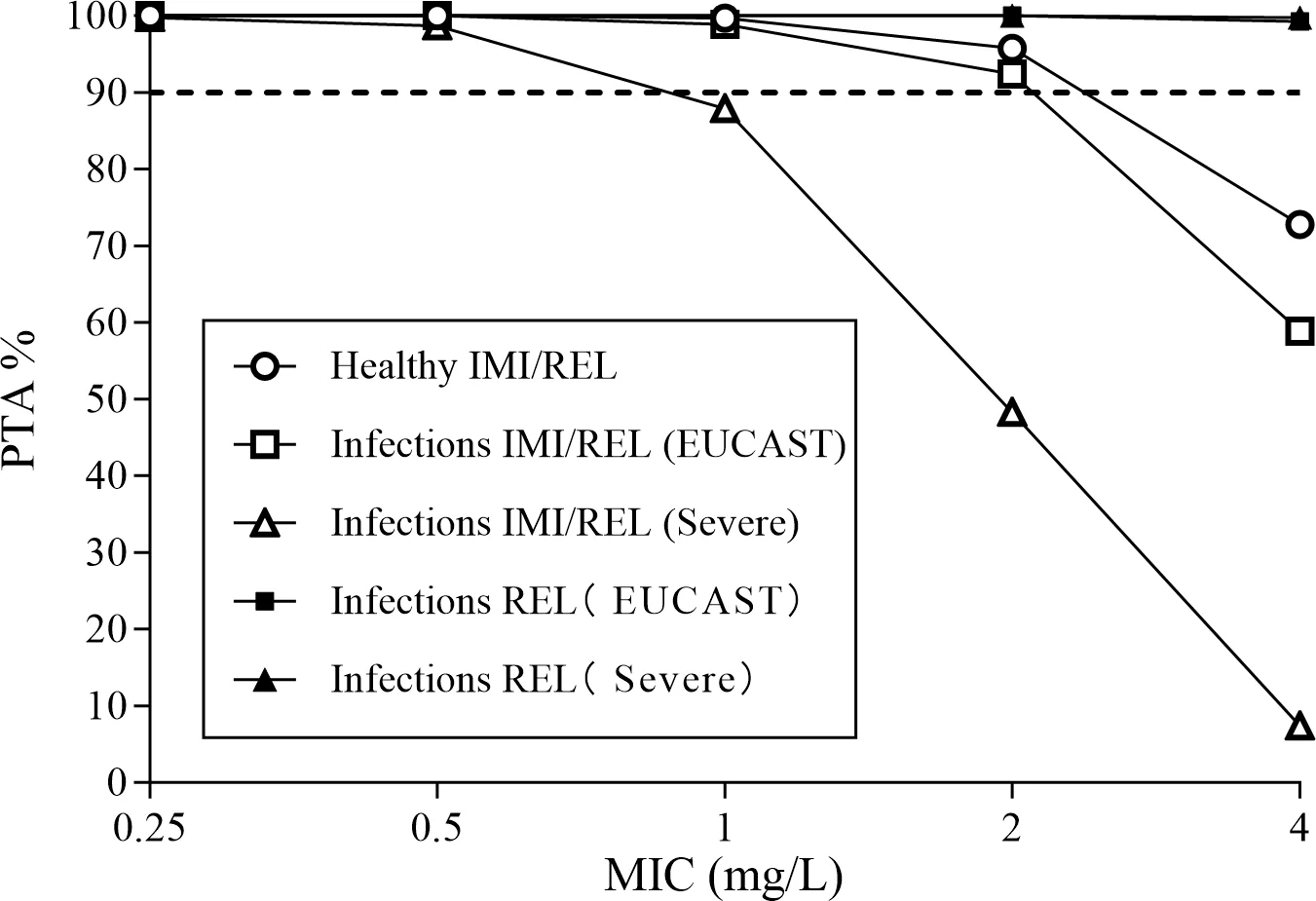
PTA for imipenem/relebactam. PTA is stratified by PK/PD targets (lower and severe criteria) and evaluated for the combination as a whole and for relebactam alone, based on the clinically recommended dose (500 mg/250 mg q6h). The dashed line indicates the 90% PTA threshold.

At the clinically recommended dosing regimen, the pharmacodynamic target attainment (PTA) for peritoneal exposure is summarized in Fig. 4. Relebactam achieved nearly 100% PTA across all the evaluated PK/PD targets. In contrast, imipenem exhibited markedly lower PTA, thereby limiting the overall pharmacodynamic performance of the combination. At an MIC of 0.5 mg/L, both simulated dosing conditions yielded high PTA. However, at an MIC of 1 mg/L, PTA declined under high-intensity PK/PD targets and no longer met the efficacy thresholds. At an MIC of 4 mg/L, the combination failed to achieve adequate PTA in any scenario.

**Fig 5:**
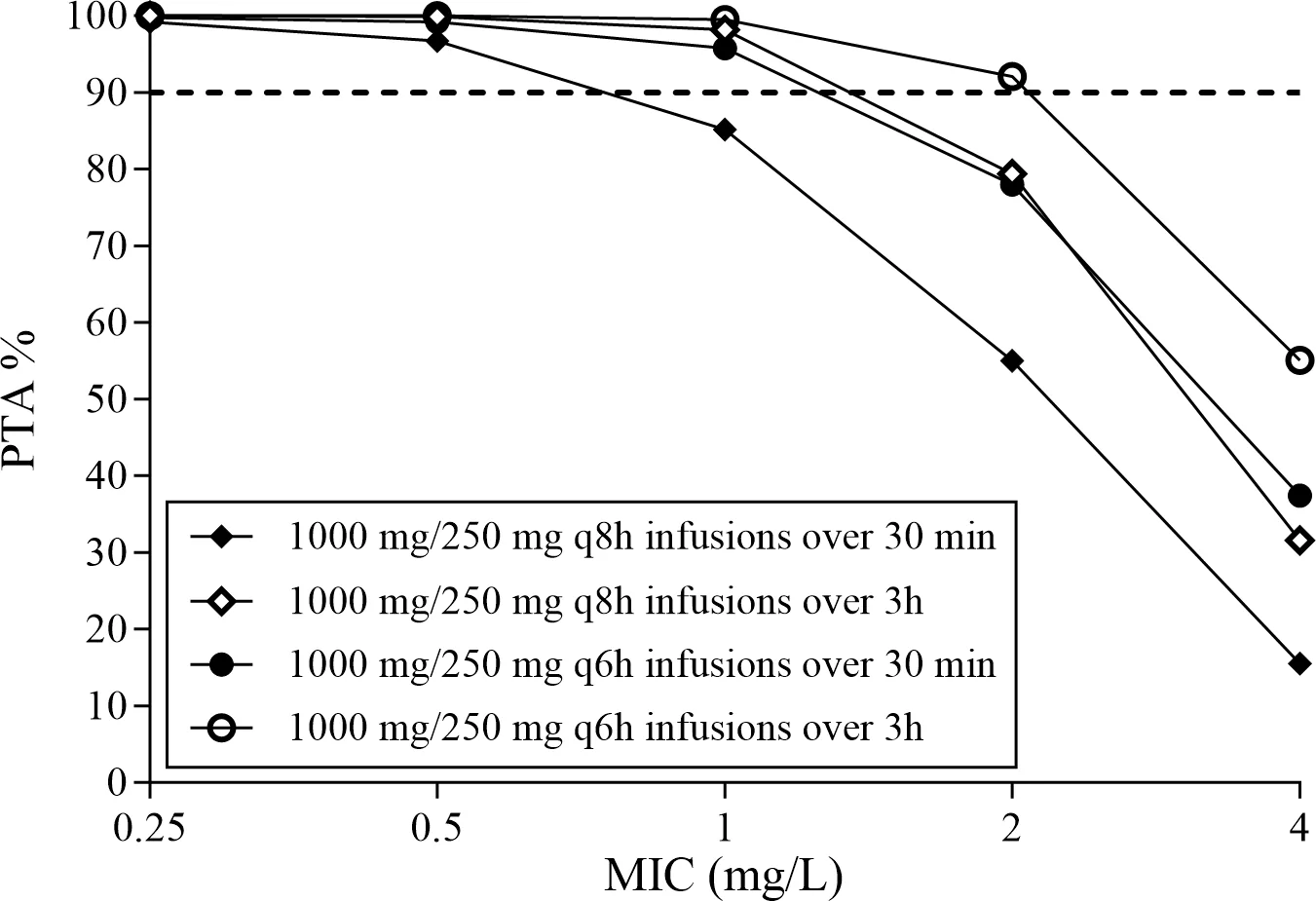
PTA for imipenem/relebactam under intensified dosing regimens (1,000 mg/250 mg, q8h and q6h, with 30 min and 3 h infusion durations), evaluated using the severe EUCAST PK/PD criteria. The dashed line indicates the 90% PTA threshold.

Given the suboptimal PTA observed at higher MIC values under high-intensity PK/PD targets, intensified regimens were further evaluated under these same targets, within the constraint of the clinically recommended maximum daily dose of imipenem (≤4 g/day; Fig. 5). Overall, when MIC ≤ 1 mg/L, all regimens achieved target PTA. At MIC ≤ 2 mg/L, extending infusion to 3 h under the 1,000 mg q6h regimen improved PTA and enabled target attainment.

## DISCUSSION

In this study, we systematically characterized the distribution of imipenem and relebactam in the peritoneal fluid of healthy and infected rats using pharmacokinetic parameters derived from unbound concentrations. To ensure stable and reproducible infection, cyclophosphamide pretreatment was introduced, as preliminary experiments showed that intraperitoneal inoculation alone did not reliably produce a consistent infection. To our knowledge, this is the first report to characterize the peritoneal pharmacokinetics of imipenem/relebactam, thereby addressing a critical gap in tissue-site data for this BL/BLI combination. Both agents exhibited rapid equilibration between plasma and peritoneal fluid, with consistent penetration ratios across time points. These findings suggest that near-instantaneous passive diffusion, without a measurable distribution delay, is the primary equilibration mechanism. Relebactam demonstrated substantially higher peritoneal penetration than imipenem. In addition, despite the fixed 2:1 dosing ratio of imipenem and relebactam, their systemic exposure did not reflect this proportion. This discrepancy can be attributed to compound-specific pharmacokinetic properties, including differences in clearance and volume of distribution, rather than analytical variability. Similar findings have been reported in clinical studies (18), indicating that dose ratios do not necessarily translate into proportional plasma exposure.

The two-compartment model is frequently used to describe the antibiotic distribution between plasma and tissues. In earlier studies (19, 20), peritoneal fluid was often treated as part of the peripheral compartment, with exposure inferred via a peritoneal-to-peripheral coefficient. This approach accounts for delayed peaks in peritoneal drug concentrations when the fluid behaves kinetically as a peripheral compartment. However, no such delay was observed in this study. Accordingly, we introduced a third compartment specifically representing the peritoneal cavity, linked to the central compartment by the plasma-to-peritoneal Kp. This structure better captured the observed rapid equilibration and produced Kp values consistent with those obtained by AUC ratio analysis.

The exposure of unbound imipenem in the peritoneal fluid (27%–29% of plasma) was substantially lower than that reported by Lefeuvre et al. (11, 12), who used cecal ligation and puncture models of peritonitis and pancreatitis. Our intraperitoneal inoculation model avoids laparotomy and associated surgical trauma, which may result in less extensive peritoneal injury and reduced drug penetration. Accordingly, this model may better represent clinical scenarios in which no surgical intervention has been performed, although it does not capture the full complexity of severe peritonitis. In patients with severe peritonitis, microdialysis studies have reported considerable variability in peritoneal-to-plasma ratios (0.265–1.125) (13), with most patients exhibiting lower peritoneal than plasma exposure. These findings highlight the influence of both experimental models and patient-specific pathophysiology on imipenem penetration. Variability in peritoneal permeability, local inflammatory responses, and potential drug degradation at the site of infection may contribute to these differences.

In contrast, relebactam achieved peritoneal fluid-to-plasma ratios of 0.81–0.86, indicating extensive penetration similar to that of other BLIs (19–22). For most BL/BLI combinations, such as aztreonam–tazobactam, ceftazidime–avibactam, and piperacillin–tazobactam, the two agents demonstrated comparable penetration, supporting balanced antimicrobial synergy at the site of infection. Therefore, our observation of discordant penetration between imipenem and relebactam is notable, and raises concerns that pharmacodynamic interactions may be compromised in the peritoneal cavity. Suboptimal exposure to imipenem relative to relebactam weakened bactericidal activity against carbapenem-resistant *Enterobacteriaceae*.

Another important finding was that infection did not significantly alter the plasma-to-peritoneal penetration ratio of either drug, suggesting preserved diffusion. However, both agents exhibited increased volumes of distribution under infectious conditions, consistent with clinical observations of altered pharmacokinetics in critically ill patients (23). These findings indicate that, although plasma concentrations may serve as a surrogate for peritoneal exposure, systemic infection can still reduce absolute drug exposure at the target site. To account for these factors, we integrated clinically derived pharmacokinetic parameters with the penetration ratios obtained in this study to perform PK/PD PTA analyses. Under the clinically recommended dosing regimen, imipenem–relebactam achieved ≥90% PTA at MIC values ≤2 mg/L based on earlier, less stringent EUCAST targets. However, when more stringent PK/PD targets were applied to reflect severe infections, PTA declined substantially, indicating a reduced likelihood of achieving effective peritoneal exposure. Under the constraint of the clinically recommended maximum dose of imipenem, when MIC = 2 mg/L, attainment of the more stringent PK/PD targets was only achieved with the 1,000/250 mg q6h regimen by extending the infusion duration to 2–3 h. However, at higher MIC values, even further intensified dosing strategies failed to achieve PK/PD targets, primarily due to the inability of imipenem to reach its pharmacodynamic targets. Therefore, for severe intra-abdominal infections, additional imipenem administration on top of the imipenem/relebactam regimen (500 mg/250 mg × four IV infusions over 30 min/3 h) may be considered to improve the likelihood of achieving PK/PD target attainment in the peritoneal compartment.

This study provides the first experimental evidence of relebactam penetration into peritoneal fluid and reveals a discordant distribution relative to its β-lactam partner. These findings highlight the importance of incorporating site-specific pharmacokinetics into dosing strategies for imipenem/relebactam in complicated intra-abdominal infections. Several limitations should be acknowledged. First, source control is a key determinant in the management of peritonitis; however, it was not incorporated into the present animal model, which may limit the ability to fully capture the impact of surgical intervention on antibiotic exposure at the site of infection. Second, although the intraperitoneal inoculation model offers good reproducibility and avoids surgical trauma, it does not fully replicate the complex pathophysiology of polymicrobial peritonitis caused by organ perforation and subsequent peritoneal contamination, particularly with respect to anaerobic involvement. Third, hemodynamic parameters were not systematically assessed, which may limit a comprehensive evaluation of infection severity. Accordingly, caution is warranted when extrapolating these findings to patients with more severe or complex clinical conditions. Further translational studies, ideally integrating PTA analyses and clinical microdialysis in patients, are needed to confirm these findings and inform optimized dosing strategies.

## MATERIALS AND METHODS

### Chemicals and reagents

Imipenem (analytical standard) was purchased from Meilunbio (Dalian, China), and relebactam was purchased from Yuanye Bio-Technology Co., Ltd. (Shanghai, China). Tazobactam, used as the internal standard, was obtained from the National Institutes of Food and Drug Control (Beijing, China). 3-(N-morpholino)propanesulfonic acid (MOPS) was obtained from Sigma-Aldrich (Darmstadt, Germany). The intravenous formulation of imipenem/cilastatin sodium used for dosing was manufactured by Merck & Co. (Elkton, USA). Formic acid (high-performance liquid chromatography [HPLC] grade) was supplied by ACS (Wilmington, USA), and acetonitrile (HPLC grade) was obtained from Merck (Darmstadt, Germany). Ultrapure water was prepared by using a laboratory purification system (Sartorius, Germany). Isoflurane and cyclophosphamide were sourced from Hengfeng Qiang Biological Technology Co., Ltd. (Haimeng, China) and cyclophosphamide (Baxter Oncology GmbH, Halle, Germany).

### Animals

Male Sprague–Dawley (SD) rats (220–250 g) were obtained from Hunan Silaikejingda Experimental Animal Co., Ltd. (Changsha, China). Animals were housed under controlled environmental conditions (22°C ± 2°C, 12 h light/dark cycle, 50%–60% relative humidity) with free access to food and water. Prior to experimentation, the rats were allowed to acclimate for 1 week to minimize stress and ensure stable physiological conditions.

### Establishment of the abdominal infection model

SD rats were randomly assigned to three groups (*n* = 6 per group): infection, vehicle control, and normal control. To suppress host immunity and facilitate infection, animals in the infection and vehicle control groups received 100 mg/kg cyclophosphamide on day 1 and 75 mg/kg cyclophosphamide on day 5 (dissolved in sterile saline). The normal control group did not receive cyclophosphamide, allowing separation of infection-related changes from the potential inflammatory effects of immunosuppression. On day 6, the rats in the infection group were intraperitoneally inoculated with 1 mL of *Klebsiella pneumoniae* suspension. The bacterial strain was cultured to logarithmic growth at 37°C with shaking (180 rpm), centrifuged, and resuspended at the target concentration. Control animals received an equal volume of sterile saline.

At 24 h post-infection, clinical signs were recorded, including activity level, posture, fur appearance, and stool consistency. Macroscopic pathological changes were examined after euthanasia. To quantify the bacterial burden, the peritoneal cavity was lavaged with 10 mL of sterile saline, and lavage fluid was aseptically collected, serially diluted, and plated on LB agar. A bacterial load >1 × 10^5^ CFU/mL was used as the threshold for successful model establishment. In addition, greater omental tissues were fixed in 4% paraformaldehyde, paraffin-embedded, and stained with HE. Histological evaluation focused on inflammatory cell infiltration, tissue integrity, and morphological changes, thereby confirming the severity of infection.

### Microdialysis probe implantation

Based on preliminary experiments confirming the successful establishment of infection, microdialysis probes were implanted into the infection and vehicle control groups during the formal study. On day 7, CMA 30 linear probes (10-mm polyamide membrane, 6 kDa molecular weight cut-off; CMA, Sweden) were activated by immersion in pure water for 30 min, followed by equilibration in sodium heparin solution (10 IU/mL). Rats were anesthetized with isoflurane using an induction chamber and maintained via a facemask (flow rate: 2.0 μL/min, concentration: 20 μg/mL) while supine on a heated surgical table. The limbs were secured with adhesive tape, and the abdominal fur was clipped and disinfected with 0.5% povidone–iodine. Two puncture sites were marked on opposite sides of the abdomen. A sterile needle was inserted through the first puncture, advanced across the peritoneum, and exited the second puncture site. The activated probe was threaded through the channel, and the needle was withdrawn while the probe was advanced to ensure that the dialysis membrane (10 mm) was fully positioned within the peritoneal cavity. Both ends of the probe were secured with sutures, and the abdominal incision was closed. The probe was connected to the microdialysis system, and its position was verified under ultrasound guidance to ensure consistent placement adjacent to the greater omentum in all animals. At the end of the experiment, probe placement was verified by necropsy. The surrounding abdominal organs and intestinal structures were systematically examined for any signs of puncture or injury. Animals with evidence of tissue damage were excluded from the analysis.

### Microdialysis experiment

Microdialysis studies were performed using the CMA 402 microdialysis system (CMA, Sweden). The *in vivo* recovery of each probe was estimated using a retrodialysis method. Briefly, Ringer’s solution without the drug was perfused through the probe at a flow rate of 2 μL/min for 60 min to establish equilibrium. The perfusion medium was replaced with Ringer’s solution containing 20 μg/mL imipenem/relebactam, and dialysates were collected every 20 min for an additional 30 min using an automated fraction collector. Probe recovery (%) was calculated using the following equation:


$$Recovery\;\left(\%\right)=\left[\frac{\left(C_{in}-C_{out}\right)}{C_{in}}\right]*100,$$

where $C_{in}$ and $C_{out}$ are the drug concentrations in the perfusate and dialysate, respectively. Mean recovery values (± SD) were determined across animals, and individual concentrations were corrected for probe-specific recovery.

For pharmacokinetic experiments, the perfusate was replaced with drug-free Ringer’s solution and flushed at 2 μL/min for 60 min to eliminate residual drugs. Imipenem (45 mg/kg) and relebactam (22.5 mg/kg) were co-administered via tail vein infusion by using a microinjection pump (Pump 11 Elite, Harvard Apparatus, USA) for 30 min. The dosing regimen was derived from the commonly used clinical dose of 500/250 mg using BSA scaling. Although this approach facilitates interspecies dose conversion, it does not fully ensure pharmacokinetic equivalence across species; therefore, additional PK/PD target attainment simulations based on clinical population pharmacokinetic (PopPK) parameters were performed to enhance translational relevance. Dialysates were collected automatically at 10 min intervals throughout the study.

Simultaneously, serial blood samples (0.2 mL) were withdrawn via a pre-implanted jugular vein catheter into heparinized tubes at baseline and at 15, 30, 40, 60, 90, 120, 150, 180, 210, and 270 min after drug administration. Samples were centrifuged at 2,000 × *g* for 10 min at 4°C to obtain plasma. After each collection, the catheter was flushed with heparinized saline to maintain its patency. To stabilize β-lactams, plasma was immediately mixed with 20% MOPS aqueous solution (9:1, [vol/vol]), and 90 μL of 2.3% MOPS solution was added to each microdialysis collection tube. Both plasma and dialysate samples were stored at −80°C until analysis.

### Sample preparation and calibration

Stock solutions of imipenem (5.0 mg/mL), relebactam (2.0 mg/mL), and the internal standard tazobactam (1.0 mg/mL) were prepared in 2% MOPS aqueous solution and stored at −80°C. Working solutions were prepared by serial dilution of either blank plasma or Ringer’s solution containing 2% MOPS to generate calibration curves and quality control (QC) samples. Calibration ranges were 0.5–100 mg/L for imipenem and 0.05–10 mg/L for relebactam. QC samples were prepared at low, medium, and high concentrations (0.8, 8, and 80 mg/L for imipenem and 0.08, 0.8, and 8 mg/L for relebactam, respectively).

Plasma samples (50 μL) were deproteinized by adding 150 μL acetonitrile and 5 μL internal standard (10 μg/mL tazobactam), vortexed, and centrifuged at 13,500 × *g* for 5 min. The resulting supernatant (100 μL) was mixed with 100 μL ultrapure water, and 30 μL was injected for analysis. For the microdialysates, 2 μL of the internal standard was added directly, and the mixture was injected without further preparation.

### Chromatographic analysis

Imipenem was quantified using an external standard method on an LC-20AD HPLC system (Shimadzu, Japan), with UV detection at 299 nm. Separation was performed on a Venusil HILIC column (4.6 × 250 mm, 5 μm; Agela Technology, China). The mobile phase consisted of 50 mmol/L potassium dihydrogen phosphate containing 0.1% formic acid and acetonitrile (30:70, [vol/vol]), delivered isocratically at 0.8 mL/min. The inter- and intra-day accuracies at four concentrations (including three QC levels and the lower limit of quantification [LLOQ]) ranged from 94.0% to 101.0%, with precision (relative standard deviation) <12.8%.

Relebactam was quantified using LC–MS/MS on a Shimadzu LCMS-8050 triple quadrupole mass spectrometer (Kyoto, Japan) coupled to an LC-30AD binary pump, SIL-20ACHT autosampler, and column oven. Separation was achieved on an Agilent Poroshell 120 EC-C18 column (3.0 × 50 mm, 2.7 μm). The mobile phase consisted of 0.1% formic acid in water (A) and 0.1% formic acid in acetonitrile (B) at a flow rate of 0.3 mL/min with the following gradient: 0–1 min, 80% B; 1–1.1 min, 80%→5% B; 1.1–5 min, 5% B. The column oven and autosampler were maintained at 40°C and 6°C, respectively.

Mass spectrometric detection was performed in positive electrospray ionization mode with optimized source parameters: desolvation line, 250°C; heating block, 400°C; interface, 300°C; nebulizing gas 3.0 L/min, drying gas 10.0 L/min, heating gas 10.0 L/min, and interface voltage 4.0 kV. Multiple reaction monitoring transitions were m/z 347.20→168.25, and m/z 298.90→138.15 for tazobactam (internal standard). Inter- and intraday accuracies ranged from 90.5% to 106.0%, with precision consistently <8.4%.

### Pharmacokinetic analysis

PK analyses were performed using Phoenix WinNonlin (version 6.0; Pharsight Co. Ltd., Missouri, USA). The peritoneal microdialysate concentrations of imipenem and relebactam represented unbound drug levels and were corrected to the actual concentrations in the peritoneal cavity based on the *in vivo* probe recovery rate. Plasma concentrations were converted to unbound values using previously reported protein-binding rates (imipenem, 20%; relebactam, 17.2%) (24).

Initial noncompartmental analysis (NCA) was conducted to estimate terminal elimination half-life (*t*_1/2_), maximum observed concentration ($C_{max}$), and the area under the concentration–time curve from zero to infinity (AUC_0–∞_). Although NCA provides descriptive PK parameters, it does not adequately capture the distribution kinetics of both agents.

Exploratory analysis indicated that the semi-logarithmic concentration–time profiles of imipenem and relebactam exhibited biphasic behavior, consistent with a two-compartment model. Moreover, rapid equilibration between the plasma and peritoneal fluid was observed without a measurable delay. Therefore, the data were further analyzed using a two-compartment structural model incorporating a peritoneal-to-plasma Kp to describe drug distribution in the peritoneal cavity.

The model was expressed using the following differential equations, representing the drug amounts in the central (Xc) and peripheral (Xp) compartments


dXc/dt=k0−k12∗Xc+K21∗Xp−K10∗Xc,

dXp/dt=k12∗Xc−K21∗Xp.

The peritoneal fluid concentration (C_peritoneal) was calculated as:


C_(peritoneal fluid)=Xc∗Kp/Vc,

where k0 is the infusion rate (mg/min, during drug administration), k12 and k21 are transfer rate constants (min⁻¹) between central and peripheral compartments, k10 is the elimination rate constant (min⁻¹) from the central compartment, Vc is the apparent central compartment volume of distribution (L/kg), and Kp is the peritoneal-to-plasma partition coefficient, defined as the ratio of unbound peritoneal fluid to unbound plasma concentrations.

### Peritoneal fluid PK/PD analysis

This study applied previously published PopPK parameters to infected patients (25). A simulation framework was developed in R (v4.5.1) using mrgsolve to generate plasma concentration–time profiles following imipenem/relebactam administration at the clinically recommended dosing regimen (500 mg/250 mg × four IV infusions over 30 min). To further evaluate pharmacodynamic target attainment under more stringent PK/PD criteria, additional intensified dosing regimens were simulated, including 1,000 mg/250 mg administered every 8 h or 6 h, with infusion durations of 30 min or prolonged up to 3 h. Plasma concentrations were converted into peritoneal fluid concentration–time curves using previously reported unbound fractions and the peritoneal penetration ratios measured in this study, thereby characterizing intraperitoneal exposure.

Two EUCAST-endorsed PK/PD thresholds were evaluated (25–27). The first set—representing the previous and less stringent EUCAST criteria—consisted of 6.5% *f*T > MIC for imipenem and a relebactam *f*AUC_0–24_/MIC ≥ 5.2. To reflect the higher pharmacodynamic requirements of severe infections and incorporate the most recent EUCAST guidance, a second, more stringent set of criteria was applied, comprising 40% *f*T > MIC for imipenem and relebactam *f*AUC_0–24_/MIC ≥ 7.5.

For each virtual subject, simulated peritoneal concentration–time profiles were assessed against the corresponding PK/PD targets. The proportion of subjects achieving each target across MIC values was then calculated to determine the probability of target attainment in infected states.
